# SPP1/OPN Alleviates Post‐Intracerebral Hemorrhage Depression and Cognitive Impairment via Nrf2/BDNF Signaling Activation in Mice

**DOI:** 10.1002/cns.70680

**Published:** 2025-12-04

**Authors:** Pengpeng Li, Yangyang Gao, Shiqing Du, Zhengqian Mu, Zhenxing Tao, Xuqi Zhang, Xudong Zhao

**Affiliations:** ^1^ Wuxi School of Medicine Jiangnan University Wuxi Jiangsu China; ^2^ Department of Neurosurgery Jiangnan University Medical Center Wuxi Jiangsu China; ^3^ Wuxi No. 2 People's Hospital, Affiliated Wuxi Clinical College of Nantong University Wuxi China; ^4^ Ningxia Medical University Yinchuan China; ^5^ Department of Neurosurgery Medical School of Nantong University, Nantong University Nantong China; ^6^ Wuxi Neurosurgical Institute Wuxi Jiangsu People's Republic of China

**Keywords:** depression, ICH, Nrf2, OPN, SPP1

## Abstract

**Background:**

Post‐stroke depression represents a prevalent neuropsychiatric complication following intracerebral hemorrhage (ICH), yet its underlying mechanisms remain less understood compared to ischemic stroke.

**Methods:**

This translational investigation employed a multi‐omics approach, combining bioinformatics analysis of depression‐related (GSE214921) and ICH‐related (GSE18193) datasets from the GEO database with experimental validation. Using a collagenase‐induced striatal ICH murine model, we evaluated the effects of intranasal administration of osteopontin (OPN, encoded by SPP1) through neurobehavioral tests, histopathological evaluation, and molecular biology techniques.

**Results:**

Integrated bioinformatics analysis identified the SPP1 signaling pathway as a potential key regulator in post‐ICH depression pathogenesis. In vivo, OPN treatment produced sustained neurobehavioral improvements at 28 days post‐ICH, significantly ameliorating neurological deficits, mitigating anxiety‐depressive behaviors, and enhancing spatial learning‐memory performance. Histopathological evaluation revealed OPN's multifaceted neuroprotective effects, including attenuated hippocampal neuroinflammation, preserved Nissl body integrity, and restored dendritic arborization complexity. Mechanistically, OPN exerted its therapeutic effects through activation of the Nrf2/BDNF signaling axis, as pharmacological inhibition of Nrf2 with ML385 completely abrogated both neuroprotection and BDNF upregulation.

**Conclusion:**

Our study is the first to demonstrate the critical role of SPP1 signaling in post‐ICH depression through modulation of the Nrf2/BDNF pathway, providing novel therapeutic targets for clinical management of this debilitating neuropsychiatric sequela.

## Graphical abstract

Intranasal osteopontin (OPN/SPP1) alleviates depression and cognitive deficits after intracerebral hemorrhage in mice. OPN activates the Nrf2/BDNF signaling pathway, reducing neuroinflammation and promoting neuronal recovery. This study identifies SPP1 signaling as a novel therapeutic target for post‐stroke neuropsychiatric sequelae.

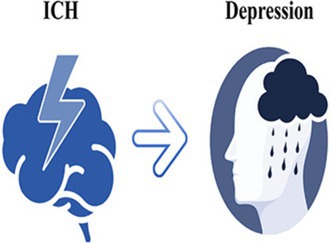

## 1 Introduction

Depression and stroke represent major contributors to global health challenges [1, 2]. The World Health Organization (WHO) highlights depression as the leading cause of disability worldwide [3], while stroke ranks as the second most common cause of mortality, the third leading contributor to disability, and a principal factor in dementia development globally [4]. Post‐stroke depression (PSD), the most prevalent neuropsychiatric complication following cerebrovascular events, imposes substantial socioeconomic burdens [5, 6]. Recent epidemiological trends reveal a surge in PSD cases corresponding with increased stroke survival rates, affecting approximately one‐third of stroke survivors [7].

Current research predominantly focuses on depression following ischemic stroke, leaving critical knowledge gaps regarding post‐intracerebral hemorrhage (ICH) depression [8]. There remains a paucity of preclinical investigations, clinical trials, and translational studies addressing this specific condition. Notably, existing stroke management guidelines lack comprehensive protocols for identifying and managing depression in ICH patients [9]. The underlying mechanisms driving post‐ICH depression also remain poorly characterized, hindering therapeutic development. Addressing these research deficiencies through targeted investigations into disease mechanisms and potential interventions could significantly improve clinical outcomes for this vulnerable patient population.

Emerging evidence highlights the multifaceted neuroprotective role of SPP1 in central nervous system recovery. Experimental findings demonstrate that SPP1 enhances necrotic debris clearance [10], mitigates neuronal apoptosis [11], preserves blood–brain barrier stability [12], and promotes both neurogenesis and angiogenesis during tissue regeneration processes [13]. Notably, recent clinical investigations revealed significant downregulation of SPP1 expression in microglia isolated from major depressive disorder (MDD) patients, correlating with impaired phagocytic capacity in these immune cells [14]. Mechanistic studies confirm SPP1's critical role in potentiating microglial phagocytosis [15]. Despite established neuroprotective effects in ischemic stroke models, the therapeutic potential and molecular pathways of SPP1 in depression following intracerebral hemorrhage remain unexplored, warranting further investigation into its clinical applications for post‐ICH neuropsychiatric complications.

The bidirectional relationship between depression and stroke is multifaceted [16]. Stroke patients exhibit a significantly higher incidence of depression compared to the general population, while individuals with depression demonstrate increased susceptibility to stroke development [17]. Both disorders share common pathological mechanisms involving inflammatory responses, mitochondrial dysfunction, and metabolic dysregulation [5]. Emerging evidence identifies six primary pathways contributing to depression pathogenesis: elevated pro‐inflammatory cytokines, activated cellular immunity, oxidative stress injury, reduced antioxidant capacity, mitochondrial impairment, and neuroprogressive changes [18].

Notably, the transcription factor Nrf2 plays a multifaceted regulatory role in depression pathophysiology and represents a promising therapeutic target [19]. Nrf2 activators may potentially modulate microglial polarization from pro‐inflammatory to anti‐inflammatory phenotypes through BDNF regulation [20]. As a critical neurotrophin implicated in mood disorders, BDNF itself has been identified as a downstream target of Nrf2 signaling [21]. Recent investigations suggest that the Nrf2/BDNF axis facilitates functional recovery in post‐stroke depression models [22].

In light of these mechanisms, our study employs an intracerebral hemorrhage (ICH) murine model to investigate SPP1's therapeutic potential in ameliorating neurological deficits, cognitive dysfunction, and affective disorders including depression and anxiety‐like behaviors. Furthermore, we aim to elucidate the involvement of the Nrf2/BDNF signaling pathway in mediating these neuroprotective effects.

## 2 Materials and Methods

### 2.1 Data Acquisition

Clinical and transcriptomic data from ICH patients were retrieved from depression‐related (GSE214921) and ICH‐related (GSE18193) datasets in the NCBI GEO repository. As this study utilized anonymized public data, no institutional review board approval or patient consent was required.

### 2.2 Differential Gene Expression Profiling

Differentially expressed mRNAs (DEGs) were identified using the limma package (v2.10) in R (v3.18.0), with significance thresholds set at |log_2_ fold‐change| > 1 and adjusted *p* < 0.05. Functional enrichment analyses were conducted to elucidate the biological relevance of DEGs.

### 2.3 ICH and Depression Related Gene Screening

ICH and depression‐associated genes were cross‐referenced using GSE214921 and intersected with GSE18193 DEGs via Venny 2.1. Overlapping genes were subjected to pathway annotation using Metascape (v3.5) for biological process enrichment, with statistical significance maintained at *p* < 0.05.

### 2.4 Integrated Bioinformatics Framework for Deciphering Molecular Networks in Post‐Hemorrhagic Stroke Depression

To investigate the pathophysiological mechanisms underlying depression following intracerebral hemorrhage, we established a comprehensive bioinformatics workflow integrating multi‐omics analytical techniques. Our investigation commenced with functional annotation of differentially expressed genes (DEGs) identified in depression‐related (GSE201332) and hemorrhagic stroke‐associated (GSE18193) transcriptomic datasets, employing Metascape's enrichment analysis suite. This initial phase delineated critical pathway disturbances implicated in post‐hemorrhagic depressive pathogenesis, revealing significant alterations in neuroinflammatory signaling and synaptic plasticity regulation [23].

For protein network characterization, we implemented a hybrid analytical pipeline combining STRING database (v11.0) interaction predictions with Cytoscape (v3.10.2)‐mediated network construction. Modular architecture analysis was performed using MCODE (v2.0.0) with optimized clustering parameters, applying connection density‐based weighting for cluster identification. Statistical validation included permutation testing (*α* = 0.05) to ensure module robustness against random network configurations [24]. This tiered analytical architecture incorporates progressive validation mechanisms, from global network reconstruction to localized cluster resolution, enhancing biological interpretability through sequential filtering of interaction data.

### 2.5 Experimental Animals and Ethical Considerations

Male C57BL/6J mice (8–12 weeks old) were housed under controlled environmental conditions (23°C ± 1°C, 40%–60% humidity) with 12‐h photoperiod cycles. Cohorts of 3–5 animals were maintained in standard cages with ad libitum access to food and water. The experimental protocol (Approval No. 2023 Y202) received institutional review board approval from The Affiliated Wuxi No. 2 People's Hospital Animal Ethics Committee, following ARRIVE reporting guidelines and national animal welfare regulations. Subject randomization was implemented through computational algorithms, with outcome assessors blinded to treatment allocations. A priori power analysis (*α* = 0.05, *β* = 0.2) determined cohort sizes, ensuring statistical validity while adhering to humane endpoint criteria.

### 2.6 Intracerebral Hemorrhage Induction Protocol

The ICH model was established through stereotaxic administration of collagenase IV (0.03 U in 2.5 μL saline) into the left striatum (depth: 3.2 mm) using a microprocessor‐controlled infusion system (0.18 μL/min). To ensure complete solution dispersion and prevent backflow, the injection cannula remained in situ for 10 min post‐infusion. Surgical closure involved bone wax application and layered suturing, with postoperative thermoregulation maintained at 37°C via heating pad support. Experimental cohorts included: sham surgery (needle insertion without collagenase), ICH control, ICH + osteopontin (OPN), and ICH + OPN + ML385 combination groups. Sham procedures replicated all surgical steps except enzymatic induction.

### 2.7 Intranasal Delivery Protocol for OPN Neuroprotection Studies

For neuroprotective evaluation, OPN was delivered via the intranasal route using nasal drops (5 μg in 50 μL total volume, 5 μL/drop). The administration protocol involved sequential instillation into alternating nostrils at 2–5 min intervals over a 20‐min period. Treatment initiation occurred 10 min following intracerebral hemorrhage (ICH) induction. OPN (5 μg in 50 μL saline) or an equal volume of saline vehicle was administered intranasally once daily until the end of the study at day 28. This methodology was designed following established protocols from prior research documenting OPN's neuroprotective efficacy in cerebral ischemia models [25].

### 2.8 Neurological Function Evaluation

To assess neurological impairment following ICH, mice were evaluated using a standardized scoring system on days 1, 3, 5, 7, 14, and 21 post‐injury. The assessment comprised six distinct components: body posture alignment, locomotor coordination, climbing proficiency, rotational tendencies, forelimb posture symmetry, and involuntary circling behavior. Each component was scored on a scale of 0–4, with 0 indicating normal function and 4 representing severe impairment. The cumulative score across all components ranged from 0 to 24, with higher values reflecting greater neurological dysfunction. This method, adapted from established protocols [26], provided a comprehensive measure of post‐ICH neurological deficits. Forelimb placement was evaluated using a whisker‐stimulated response test. Mice were positioned at the edge of a flat surface, and one side of their whiskers was gently stimulated. Healthy mice promptly placed the opposite forelimb onto the surface. The test measured the success rate over 10 attempts. In the corner navigation test, mice were guided into a 30‐degree angled corner and allowed to choose their turning direction to exit. The direction of each turn was recorded across 10 trials, and the proportion of left turns was determined [26].

### 2.9 Spatial Learning and Memory Assessment

#### 2.9.1 Morris Water Maze (MWM)

The Morris water maze (MWM) was utilized to evaluate spatial learning and memory capabilities in rodents. The experimental setup included a circular black pool (120 cm in diameter) filled with water, with a hidden escape platform (10 cm in diameter) positioned 0.5 cm below the water surface in one of four designated quadrants. Behavioral data were collected using the SMART 3.0 animal behavior analysis system (Panlab, Spain). During the training phase, mice were introduced into the pool from random starting points along the wall and allowed to locate the submerged platform. Training sessions consisted of one trial per day for four consecutive days, beginning on day 24 post‐ICH. On day 28, spatial memory was assessed by analyzing navigation patterns and trajectory parameters during the testing phase, providing insights into cognitive function and memory retention.

### 2.10 Depression‐Like Behavior

#### 2.10.1 Forced Swim Test (FST)

To evaluate depression‐like behaviors, the forced swim test (FST) was employed [27]. The experimental setup consisted of a transparent cylindrical container (height: 20 cm, diameter: 22 cm) filled with water to a depth of 10 cm, maintained at a temperature of 24°C ± 1°C. On day 28 post‐ICH, each mouse was placed in the water and allowed to swim for a total of 6 min. The test focused on the final 4 min of the session, during which immobility time was recorded. Immobility was defined as the absence of active movement, with only minimal motions to keep the head above water. The duration of immobility served as an indicator of depression‐like behavior, with longer immobility times reflecting greater behavioral despair.

#### 2.10.2 Tail Suspension Test (TST)

On day 28 post‐ICH, TST was used to assess depression‐like behavior [28]. Mice were acclimatized to the testing room for 1 h. Each mouse was suspended by the tail 55 cm above the floor using adhesive tape, with a small tube placed around the tail to prevent climbing. Behavior was recorded for 6 min, and immobility time (absence of movement) was measured as an indicator of behavioral despair.

#### 2.10.3 Sucrose Preference Test (SPT)

On day 25, individual mice were housed in cages equipped with two bottles—one containing plain water and the other a 1% sucrose solution. Initial weights of both bottles were recorded, and their positions were alternated daily. By day 28, the bottles were reweighed to determine the volume of liquid consumed. Sucrose preference was expressed as a percentage, calculated by dividing the amount of sucrose solution consumed by the total liquid intake: sucrose preference (%) = sucrose consumption (g)/[water consumption (g) + sucrose consumption (g)] × 100.

### 2.11 Anxiety‐Like Behavior

#### 2.11.1 Open Field Test

On the 28th day, the open field test was conducted to evaluate locomotor activity and anxiety‐related behaviors [29]. Before the experiment, mice were allowed to adjust to the testing environment for 2 h. Each mouse was then gently placed in the center of the open field apparatus and allowed to explore freely for 5 min. To eliminate potential odor cues, the apparatus was thoroughly cleaned with alcohol after each trial. Movement patterns were recorded and analyzed using automated video tracking software. Parameters such as the duration and distance traveled in the central and peripheral zones of the apparatus were measured to assess behavioral responses.

### 2.12 Western Blot

On day 28, Western blot analysis was conducted to assess the expression levels of Nrf2 and BDNF proteins in brain tissues surrounding the injury site. Brain samples were homogenized on ice using a cold lysis buffer (RIPA:PMSF = 100:1) supplemented with a protease inhibitor cocktail. Protein concentrations were quantified using a BCA assay kit. Equal amounts of protein (35 μg per sample) were separated by SDS‐PAGE and transferred onto nitrocellulose membranes. The membranes were blocked with nonfat milk for 2 h and then incubated overnight at 4°C with primary antibodies targeting Nrf2 (1:1000; CST), BDNF (1:500; Abcam), and GAPDH (1:10,000; Proteintech). Following primary antibody incubation, the membranes were treated with HRP‐conjugated secondary antibodies (1:10,000; Proteintech) at room temperature for 2 h. Protein bands were visualized using a FluorChem imaging system with enhanced chemiluminescence (ECL). Band intensities were analyzed and normalized to the loading control using ImageJ software.

### 2.13 Immunofluorescence Staining

On day 28, mice from each group were deeply anesthetized with 10% chloral hydrate and transcardially perfused with saline, followed by 4% paraformaldehyde. Brains were carefully dissected, post‐fixed in 4% paraformaldehyde for 24 h, and sequentially dehydrated in 20% and 30% sucrose solutions. Tissue sections of 20 μm thickness were prepared using a freezing microtome (Leica CM1950, Germany). Sections were blocked with 5% goat serum in phosphate‐buffered saline (PBS) containing 0.1% Triton‐X 100 for 2 h at room temperature. Primary antibody incubation was performed overnight at 4°C using rabbit anti‐BDNF (1:500; Abcam). After washing, sections were incubated with goat anti‐rabbit IgG secondary antibody (1:100; CST) for 2 h at 37°C. Nuclei were counterstained with 4′,6‐diamidino‐2‐phenylindole (DAPI, 1:1000; Sigma). Fluorescence images were captured using a fluorescence microscope (Olympus Corporation, Japan). For quantification, four fields per section and five consecutive sections were analyzed. The number of BDNF, Nrf2‐positive cells in each field was counted, and the average value from 20 fields was calculated to represent BDNF, Nrf2 expression in each mouse.

### 2.14 Golgi Staining and Analysis of Dendritic Spine Densities

Mice from all experimental groups were anesthetized using isoflurane and perfused with 350–500 mL of warm saline (37°C) containing 0.5% sodium nitrite, followed by 500 mL of 4% formaldehyde for 1–2 h. Subsequently, the rats were treated with 500 mL of mordant dye, which included 5% chloral hydrate, 5% potassium dichromate, and 4% formaldehyde, for 3–4 h. The brains, including the hippocampus, were carefully extracted and immersed in the mordant dye for 4 days in a dark environment. Afterward, the tissues were transferred to a 1.5% silver nitrate solution, which was replaced daily, for an additional 3 days. Finally, the brain tissues were sectioned into 35‐μm slices using an EM UC7 ultramicrotome (Leica, Germany), fixed, and imaged. Dendritic spine densities were quantified using ImageJ software (NIH, USA).

### 2.15 Statistical Analysis

The data were presented as means ± standard deviation (SD), dot plots, or bar graphs. The normality of the data distribution was assessed using the Shapiro–Wilk test to determine the appropriate statistical method. Mortality rates were compared using the chi‐squared test. For comparisons between two groups, a two‐tailed Student's *t*‐test was applied if the data followed a normal distribution. Differences among multiple groups were analyzed using one‐way or two‐way ANOVA, followed by Bonferroni post hoc tests for pairwise comparisons. A *p*‐value of less than 0.05 was considered statistically significant. All analyses were performed using GraphPad Prism software (version 5.0; GraphPad Software Inc., La Jolla, CA). Outliers were identified based on a normal distribution assumption, with data points exceeding twofold the standard deviation from the mean being excluded.

## 3 Result

### 3.1 Screening for Hub Genes in Cerebral Hemorrhage‐Associated Depression

**FIGURE 1:**
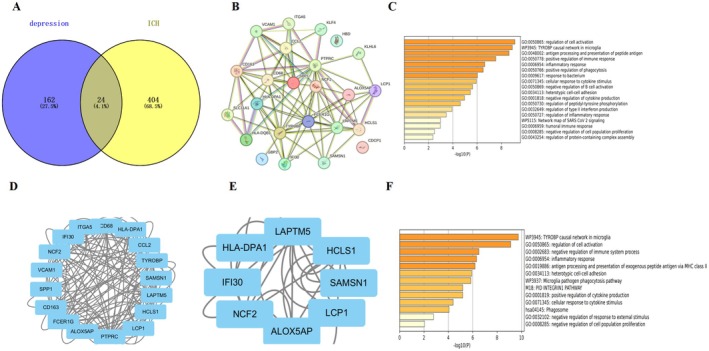
(A) The Venn diagram illustrates the intersection of differentially expressed genes. We integrated the data from depression‐related (GSE214921) and ICH‐related (GSE18193) datasets to assess the DEGs. (B) This step fortified the network of the associated provisions. (C) A Metascape analysis produced a bar graph depicting 20 biological pathways prioritized by P‐values and gene percentages (statistical significance, *p* < 0.01). The findings point to substantial enrichment in biological processes associated with the response to regulation of cell activation. (D) The Molecular Complex Assay algorithm identified three key modules for network gene clustering: Cluster 1. (E) Cluster 2. (F) A Metascape analysis produced a bar graph depicting 20 biological pathways prioritized by *p*‐values and gene percentages (statistical significance, *p* < 0.01). The findings point to substantial enrichment in biological processes associated with the response to the TYROBP casual network in microglia.

To investigate the core genes associated with post‐intracerebral hemorrhage (ICH) depression, researchers analyzed microarray datasets from depression‐related (GSE214921) and ICH‐related (GSE18193). Differential expression analysis using GEO2R identified 162 depression‐related differentially expressed genes (DEGs) and 404 ICH‐related DEGs. The intersection of these datasets revealed 24 overlapping DEGs potentially implicated in post‐ICH depression (Figure 1A,B).

Metascape pathway enrichment analysis demonstrated significant involvement of these DEGs in critical biological processes, including regulation of cell activation and TYROBP causal networks in microglia (Figure 1C). Protein–protein interaction (PPI) network construction via STRING database (interaction score > 0.4) followed by MCODE clustering in Cytoscape identified 14 hub genes forming a key functional module (Figure 1D,E). Functional annotation of cluster 1 revealed predominant association with interleukin‐4/13 signaling, extracellular matrix organization, and regulation of wound response pathways (Figure 1F).

Notably, SPP1 and its interaction partners CD44 and ITGB2 emerged as priority candidates. This selection was further supported by postmortem evidence showing significantly reduced SPP1 expression in the occipital cortex gray matter of major depressive disorder (MDD) patients compared to controls [14]. These findings highlight SPP1 as a promising therapeutic target warranting mechanistic investigation in post‐ICH depression pathogenesis.

### 3.2 Effect of OPN on Mortality After ICH

The effect of OPN on post‐intracerebral hemorrhage mortality was analyzed through comparison of mortality rates between the control ICH group (5/33, 15.1%) and the OPN‐treated ICH group (3/35, 8.5%). Although the OPN intervention group demonstrated a non‐significant reduction in mortality compared to the control cohort, this difference did not reach statistical significance (*p* > 0.05, Figure S1).

### 3.3 OPN Alleviates the Neurological Deficit Score Following Intracerebral Hemorrhage

**FIGURE 2:**
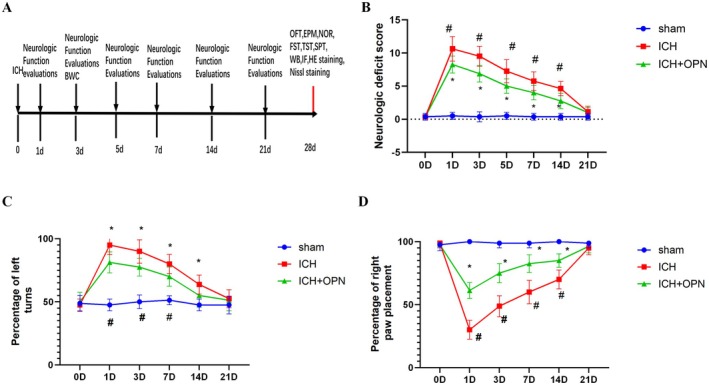
(A) Schematic representation of the experimental design. (B) OPN demonstrates neuroprotective effects in post‐ICH functional recovery. Intervention significantly attenuated neurological impairment scores in ICH mice at post‐operative days 3, 5, 7, and 14 compared to standard housing conditions (*n* = 8/group; Statistical significance is denoted as follows: *p < 0.05, ICH versus sham, ^#^ *p* < 0.05, ICH versus ICH + OPN; repeated measures ANOVA with Bonferroni correction). (C) Behavioral assessment using the corner turn test revealed enhanced directional preference in ICH + OPN mice relative to ICH counterparts at days 3, 5, and 7 post‐ICH (*n* = 8/group; statistical significance is denoted as follows: *p < 0.05, ICH versus sham, ^#^ *p* < 0.05, ICH versus ICH + OPN; repeated measures ANOVA with Bonferroni correction). (D) Motor performance scores showed statistically significant improvements from day 3 through day 14 post‐ICH (*n* = 8/group; Statistical significance is denoted as follows: *p < 0.05, ICH versus sham, ^#^ *p* < 0.05, ICH versus ICH + OPN; repeated measures ANOVA with Bonferroni correction).

Following induction of intracerebral hemorrhage (ICH), both experimental groups exhibited a significant elevation in neurological deficit scores on postoperative day 1 (POD1), with subsequent progressive improvement over time (Figure 2A). Notably, comparative analysis revealed that the OPN‐treated ICH group demonstrated substantially better functional outcomes compared to the untreated ICH controls. This therapeutic advantage was statistically significant at all measured time points: days 1 (*p* < 0.05), 3 (*p* < 0.01), 5 (*p* < 0.01), 7 (*p* < 0.05), and 14 (*p* < 0.05) post‐surgery (*n* = 8 per group; Figure 2B). The persistent between‐group differences throughout the observation period suggest a sustained neuroprotective effect of OPN administration.

OPN treatment significantly enhanced left‐turn performance in the corner turn test at multiple time points following intracerebral hemorrhage (ICH). The percentage of left turns showed marked increases on postoperative days 3 (*p* < 0.01), 5 (*p* < 0.05), and 7 (*p* < 0.05) compared to control groups, with 8 mice analyzed per experimental group (Figure 2C). Furthermore, the therapeutic intervention demonstrated sustained benefits in forelimb motor function, as evidenced by significant improvements in motor performance scores from day 3 through day 14 post‐ICH. Statistical significance was achieved at day 3 (*p* < 0.01), day 5 (*p* < 0.05), day 7 (*p* < 0.05), and day 14 (*p* < 0.05), with consistent group sizes maintained throughout the observation period (*n* = 8 per group; Figure 2D).

### 3.4 OPN Alleviates Depression‐Like Behaviors Following ICH

**FIGURE 3:**
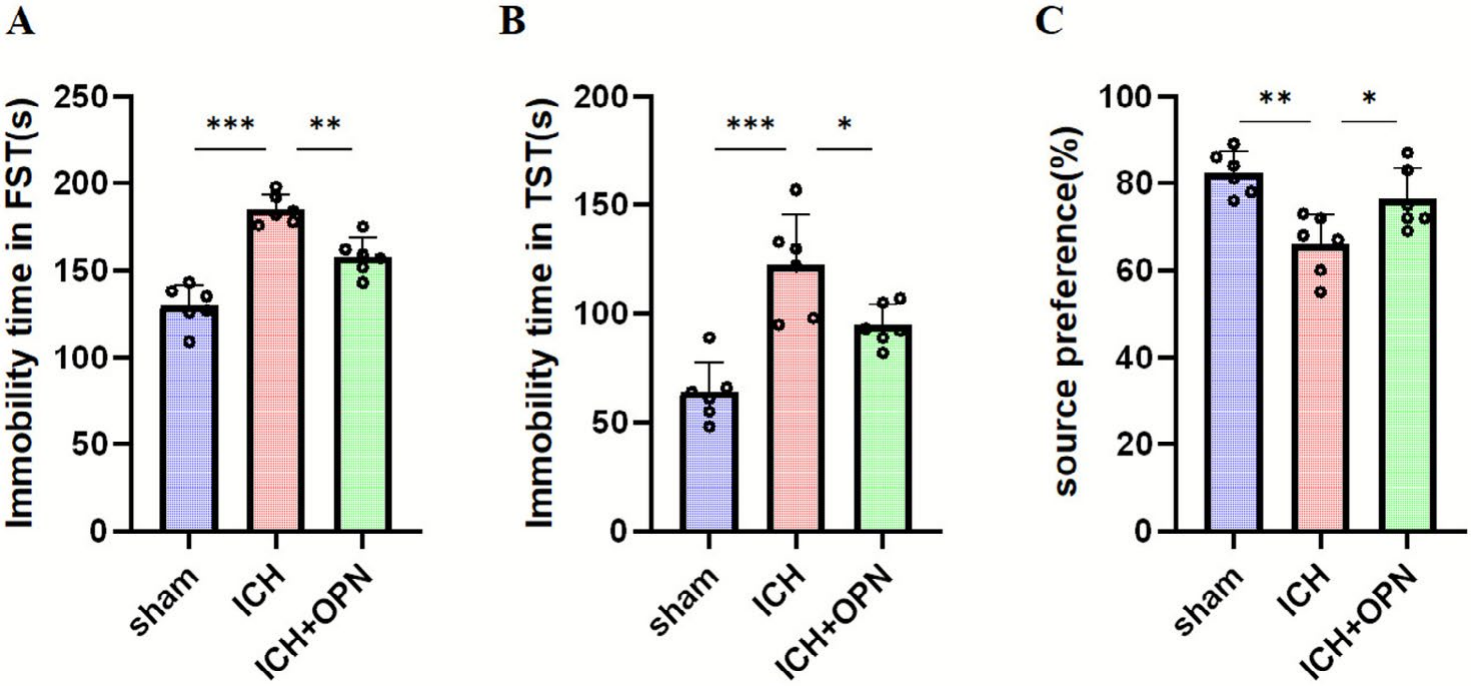
(A, B) Compared to the ICH group, mice receiving OPN supplementation displayed significantly decreased immobility durations during both the forced swim test and tail suspension test at day 28 post‐intervention (*n* = 8/group). Statistical significance: *p < 0.05, **p < 0.01, ***p < 0.001 (one‐way ANOVA or Kruskal–Wallis test post Bonferroni correction, following assessment for normality using the Shapiro–Wilk test). (C) In sucrose preference evaluations, OPN‐treated ICH mice consumed significantly greater volumes of sucrose solution than untreated ICH counterparts by day 28 (*n* = 8/group; *p < 0.05, **p < 0.01; one‐way ANOVA or Kruskal–Wallis test post Bonferroni correction, following assessment for normality using the Shapiro–Wilk test).

Behavioral assessments revealed significant therapeutic effects of OPN administration. In the forced swim test (FST), mice receiving OPN treatment after ICH demonstrated significantly reduced immobility time compared to the ICH control group at day 28 (FST: *p* < 0.01, *F* = 20.59; TST: *p* < 0.05, *F* = 18.68; each group *n* = 6; Figure 3A,B). The tail suspension test (TST) results corroborated these findings, showing decreased immobility duration in the treatment group. Furthermore, sucrose preference test (SPT) measurements indicated enhanced hedonic responsiveness in OPN‐treated animals, with significantly higher sucrose solution consumption compared to the ICH + SE group (*p* < 0.05, *F* = 22.87; each group *n* = 6; Figure 3C).

### 3.5 OPN Reduces Anxiety‐Like Behavior Following ICH

**FIGURE 4:**
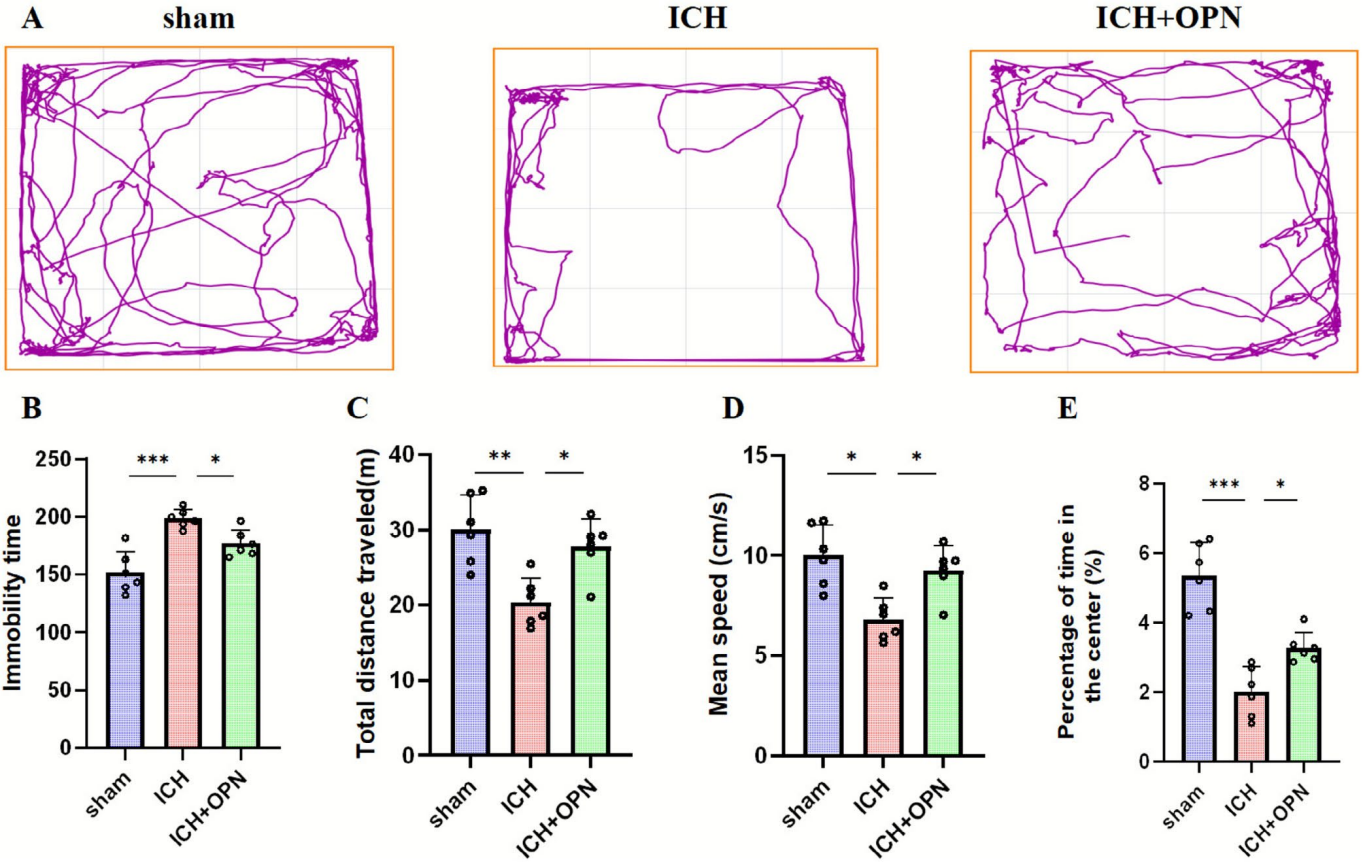
(A) Schematic diagram of movement trajectories in the open field test for three groups of mice on postoperative day 28. (B) Compared with the ICH group, the ICH + OPN group significantly reduced resting time. (C) Total travel distance of mice in the open field test among groups. (D) Comparison of movement speed among groups in the open field test. (E) Percentage of time spent in the central area. Data were analyzed using one‐way ANOVA or Kruskal–Wallis test post Bonferroni post hoc test, following assessment for normality using the Shapiro–Wilk test (*p < 0.05, **p < 0.01, ***p < 0.001).

In the open field test, ICH mice exhibited significantly reduced activity in the central zone compared to sham controls (Figure 4A). OPN administration effectively reversed this behavioral alteration. Quantitative analysis revealed three key findings: First, the ICH group demonstrated prolonged immobility time compared to both sham and ICH + OPN groups (*p* < 0.001 vs. sham; *p* < 0.05 vs. ICH + OPN; *F* = 18.75; *n* = 6/group; Figure 4B). Second, locomotor assessment showed that ICH mice had significantly lower average velocity than sham‐operated and OPN‐treated animals (*p* < 0.01 vs. sham; *p* < 0.05 vs. ICH + OPN; *F* = 10.16; Figure 4D). Third, at day 28 post‐ICH, OPN treatment significantly improved exploratory behavior as evidenced by increased total travel distance (*p* < 0.01; *F* = 10.16) and higher percentage of time spent in the central zone (*p* < 0.001; *F* = 31.74) compared to untreated ICH mice (*n* = 6/group; Figure 4C,E).

### 3.6 OPN Improves Spatial Learning and Memory Function Following ICH

**FIGURE 5:**
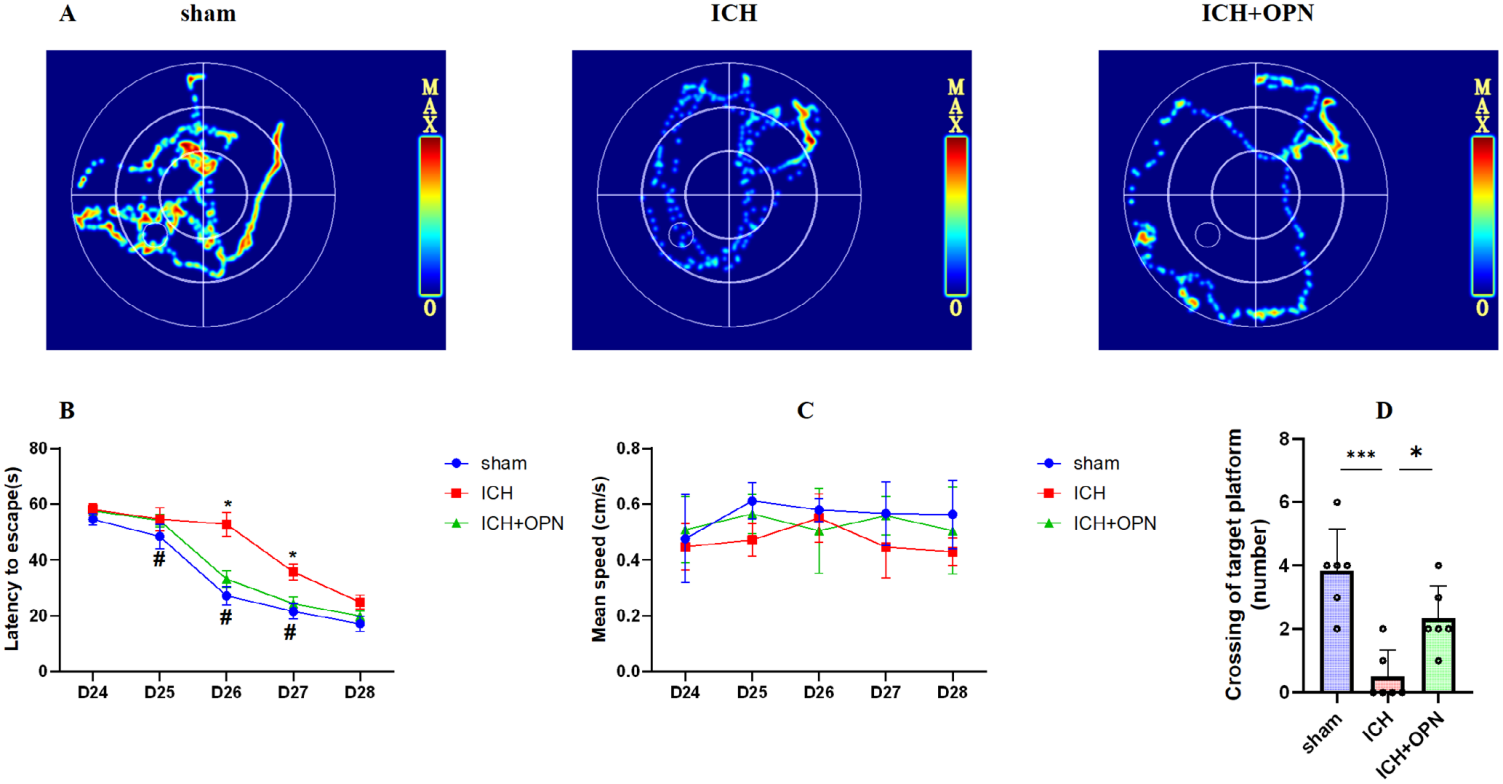
(A) Schematic diagrams of swimming trajectories in the Morris Water Maze (MWM) test for each mouse group on day 28. (B) MWM testing demonstrated that compared with the ICH group, the ICH + OPN group exhibited significantly shortened escape latencies on days 26, 27, and 28 (*n* = 6 per group; *p < 0.05 versus sham group; ^#^ *p* < 0.05 versus ICH + SE group; analyzed by repeated measures ANOVA with Bonferroni post hoc tests). (C) OPN showed no significant effect on average swimming velocity during days 25–28 (*p* > 0.05 vs. ICH group; repeated measures ANOVA with Bonferroni correction, *F* = 2.12). (D) On day 28, the ICH + OPN group displayed significantly more platform crossings compared to the ICH group (*n* = 6 per group; *p < 0.05, ***p < 0.001; analyzed by one‐way ANOVA or Kruskal‐Wallis test post Bonferroni post hoc test, following assessment for normality using the Shapiro–Wilk test).

As depicted in Figure 5A, representative swimming trajectories from different experimental groups during the Morris water maze (MWM) test demonstrate distinct spatial search patterns. In the hidden platform acquisition trials, sham‐operated mice exhibited superior performance compared to the intracerebral hemorrhage (ICH) group across training days 25–27 (*p* < 0.05 for all comparison days, *n* = 6; Figure 5B), indicating significant impairment of spatial learning and memory functions following ICH induction. Notably, osteopontin (OPN) administration effectively mitigated these cognitive deficits. Critical analysis of locomotor parameters revealed comparable swimming velocities among all three groups throughout the testing period (*n* = 6; Figure 5C), ruling out potential confounding effects of motor impairments on task performance.

The probe trial analysis further corroborated these findings. The ICH group showed markedly reduced platform crossings compared to sham controls (*p* < 0.001, *n* = 6, *F* = 14.12; Figure 5D), a deficit that was substantially ameliorated by OPN intervention (*p* < 0.05). This multiparametric assessment confirms that OPN treatment preserves essential components of spatial navigation while maintaining baseline locomotor function in the ICH model.

### 3.7 OPN‐Mediated Promotion of Nrf2 and BDNF Expression and Its Neuroprotective Effects Are Reversed by ML385

**FIGURE 6:**
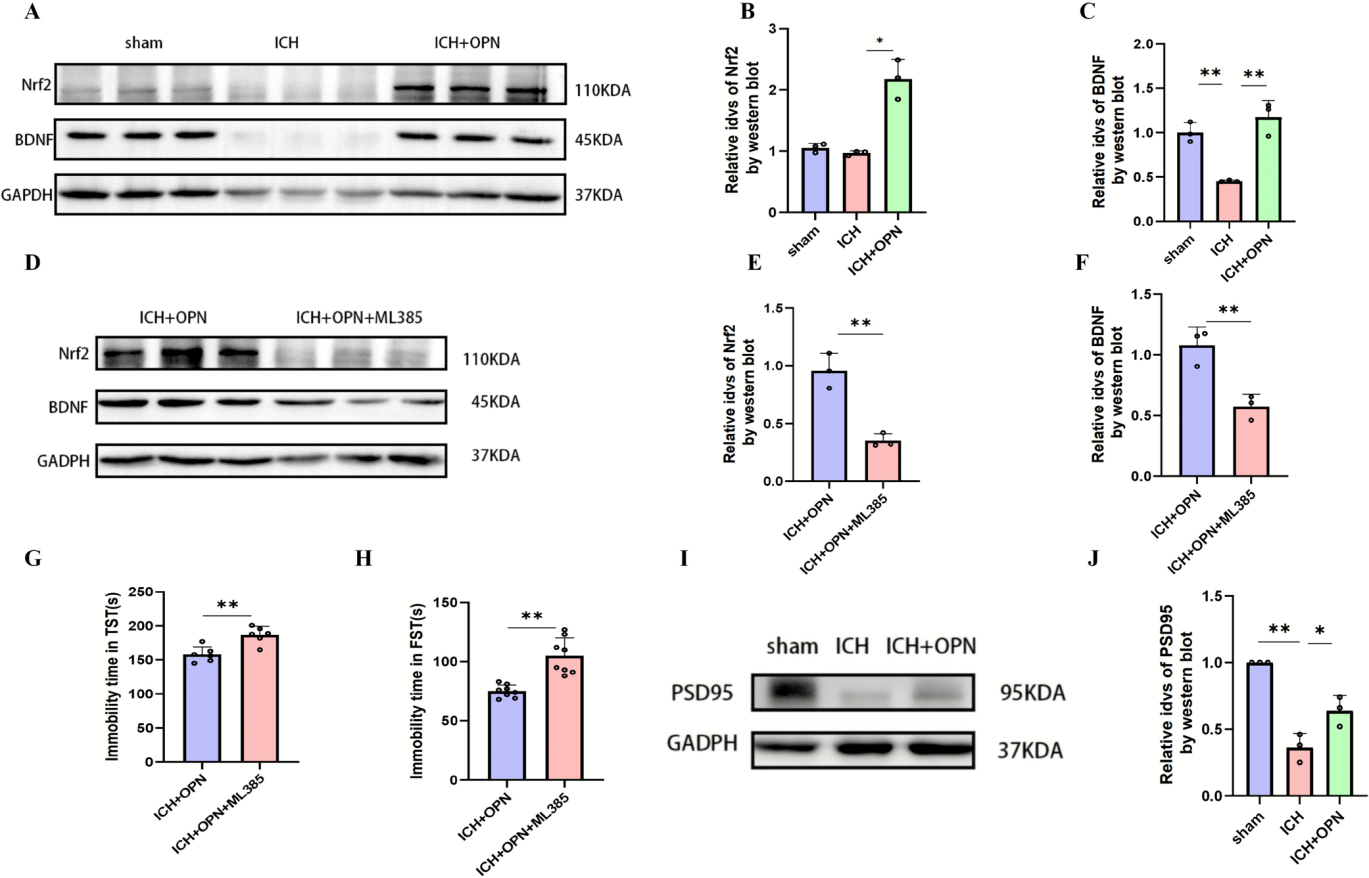
(A–C) Western blot analysis of Nrf2 and BDNF expression in brain tissue lysates from intracerebral hemorrhage (ICH) and osteopontin (OPN)‐treated ICH (OPN + ICH) groups. Nrf2 and BDNF levels were significantly elevated in the OPN + ICH group compared to the ICH group, suggesting OPN enhances Nrf2/BDNF pathway activation. Data: Mean ± SEM (*n* = 3). Statistics: One‐way ANOVA with Tukey's post hoc test; **p < 0.01, *p < 0.05 versus ICH. (D–F) Western blot analysis of Nrf2 and BDNF expression in ICH + OPN and ICH + OPN + ML385 (Nrf2 inhibitor) groups. ML385 administration reduced Nrf2 and BDNF levels compared to the ICH + OPN group. Data: Mean ± SEM (*n* = 3). Statistics: Unpaired *t*‐test; **p < 0.01. (G, H) Compared to the ICH + OPN group, mice receiving ML385 supplementation displayed significantly prolonged immobility durations during both the forced swim test (G) and tail suspension test (H) at day 28 post‐intervention (*n* = 8/group). Statistics: Unpaired *t*‐test; **p < 0.01. (I, J) Western blot analysis of PSD95 expression in brain tissue lysates from ICH and OPN‐treated ICH (OPN + ICH) groups. PSD95 levels were significantly elevated in the OPN + ICH group compared to the ICH group (J). Data: Mean ± SEM (*n* = 3). Statistics: One‐way ANOVA with Tukey's post hoc test; **p < 0.01, *p < 0.05 versus ICH.

Western blot analysis revealed that at day 28 post‐induction, the ICH + OPN group exhibited marked upregulation of Nrf2 and BDNF protein expression compared to the ICH group (*p* < 0.05, *F* = 32.58 for Nrf2; *p* < 0.01, *F* = 26.63 for BDNF; *n* = 3 per group; Figure 6A–C). However, co‐administration of the Nrf2 inhibitor ML385 effectively abolished these OPN‐mediated effects, significantly suppressing both Nrf2 and BDNF expression levels (*p* < 0.01 for both comparisons; *n* = 3; Figure 6D–F). Behavioral assessments demonstrated that ML385 administration notably prolonged immobility duration in both forced swim and tail suspension tests (*p* < 0.01 for both paradigms; *n* = 6; Figure 6G,H).

**FIGURE 7:**
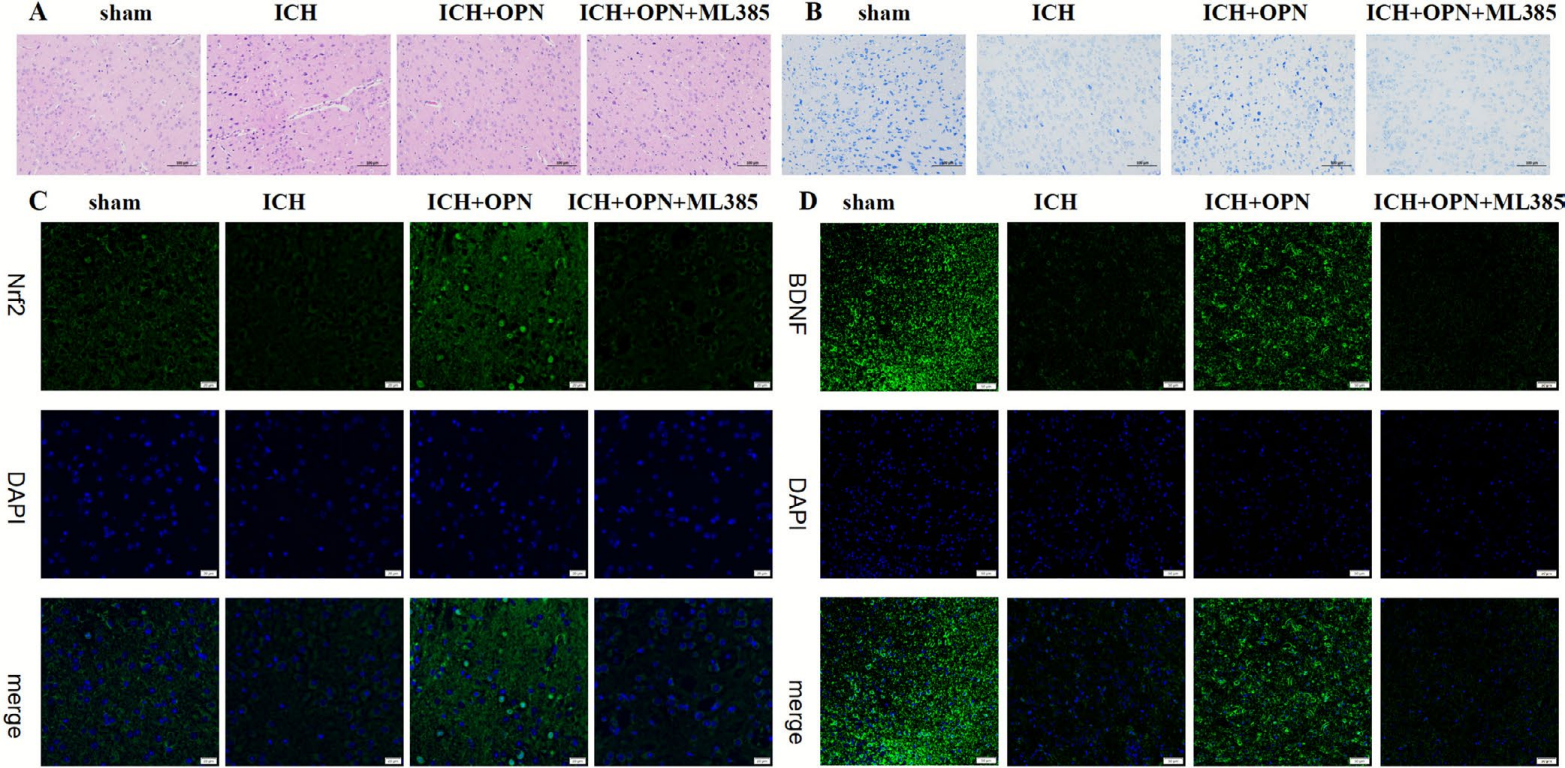
(A) HE staining reveals nuclear condensation (black arrows) and vacuolar degeneration (arrowheads) in ICH and ICH + OPN + ML385 groups, with partial structural preservation in OPN‐treated specimens. (B) Nissl staining demonstrates disrupted Nissl body integrity in injury models (dashed circles), while OPN intervention shows improved Nissl substance retention. Scale bars: 100 μm. (C) Immunofluorescence staining of Nrf2 in the ICH + OPN group showed increased fluorescence intensity and pronounced nuclear translocation compared to ICH controls. (D) BDNF immunofluorescence intensity was significantly elevated following OPN treatment. Both effects were fully reversed by co‐treatment with ML385, a selective Nrf2 inhibitor, demonstrating the pivotal role of Nrf2 signaling in mediating OPN‐induced neuroprotection.

Immunofluorescence staining further corroborated these findings. Enhanced Nrf2 fluorescence intensity with pronounced nuclear translocation was observed in the ICH + OPN group compared to ICH controls (Figure 7C). Similarly, BDNF immunofluorescence showed significant intensification following OPN treatment (Figure 7D). These morphological changes were completely reversed by ML385 co‐treatment, confirming the critical role of Nrf2 signaling in mediating OPN's neuroprotective effects. Building upon our previous findings that demonstrated OPN's ability to activate the Nrf2 pathway and upregulate downstream antioxidants including HO‐1, the current study focuses on investigating the novel role of the Nrf2‐BDNF axis in mediating OPN's effects on post‐ICH depression [30].

Hemorrhagic stroke and depression are frequently associated with marked histomorphological alterations characterized by extensive cellular edema and necrosis. To visualize hippocampal histological changes, hematoxylin–eosin (HE) staining and Nissl staining were performed, revealing distinct morphological differences across experimental groups (Figure 7A,B). Specifically, the ICH and ICH + OPN + ML385 groups exhibited neuronal pyknosis and vacuolation. In contrast, OPN‐treated rats showed mitigated tissue damage with reduced nuclear condensation and mild connective tissue loosening. Sham‐operated controls maintained dense connective architecture without pathological changes. Nissl staining demonstrated intact polygonal Nissl bodies in normal neurons, whereas injured neurons displayed diminished Nissl substance with structural dissolution. The OPN treatment group exhibited preserved neuronal morphology with near‐normal Nissl body dimensions and density, despite partial residual dissolution, contrasting with the pronounced Nissl depletion observed in the ICH and combination treatment groups.

### 3.8 OPN Mitigates ICH‐Induced Reductions in Dendritic Complexity and Spine Density

**FIGURE 8:**
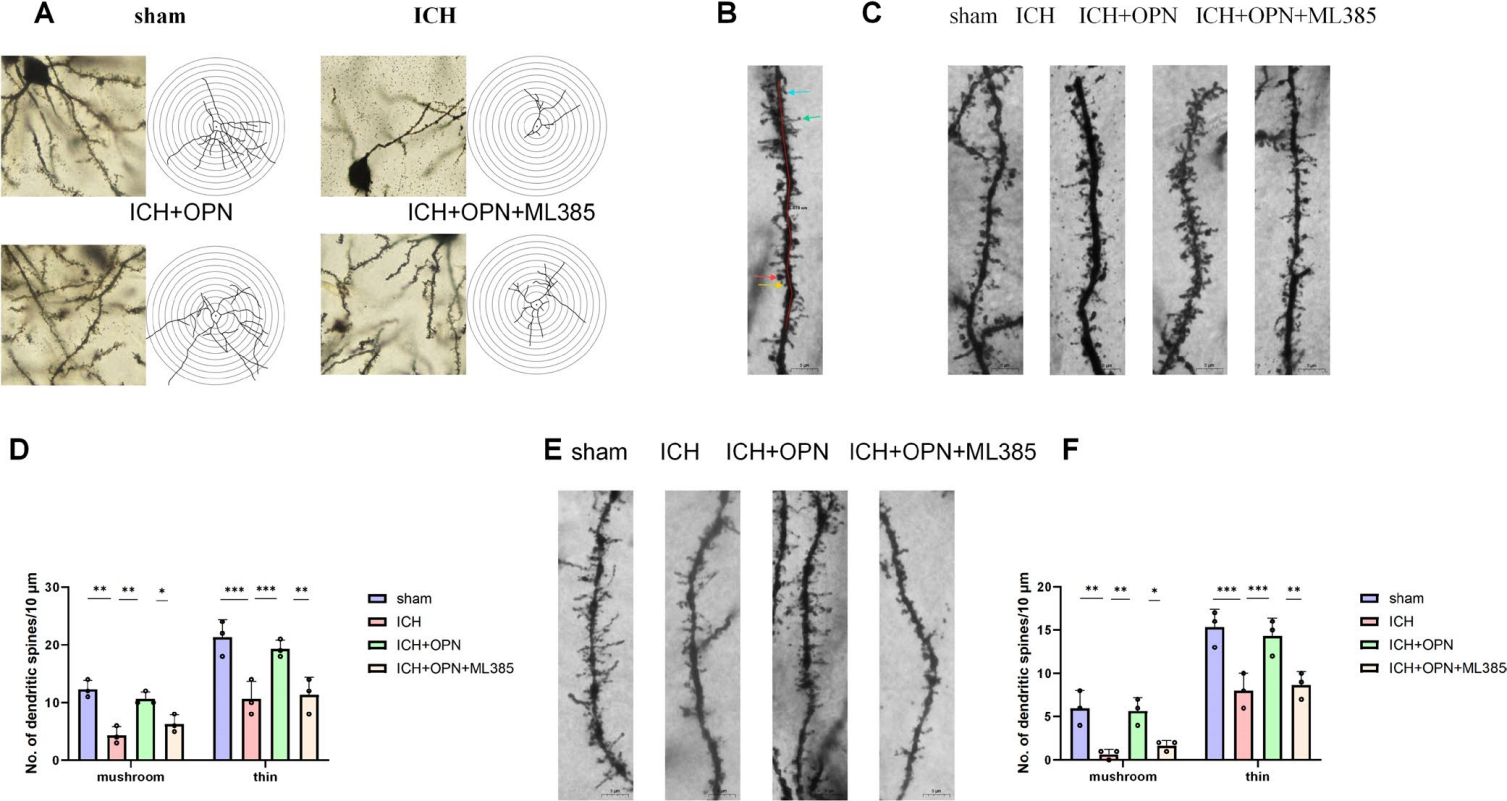
(A) Prefrontal cortex neuronal dendrites were examined using Golgi staining. (B) Spine quantification was performed on complete neurons in 1000× images, counting mushroom (red arrow), stubby (yellow arrow), thin (blue arrow), and filopodia‐like (green arrow) spines within 30–90 μm of secondary dendrites. (C, D) Spine density in prefrontal cortex pyramidal cells. (E, F) Spine density in CA1 pyramidal cells. Data: Mean ± SEM. *p < 0.05, **p < 0.01, ***p < 0.001; *n* = 3 per group (one‐way ANOVA or Kruskal–Wallis test post Bonferroni/Dunn's test, following assessment for normality).

Neuronal dendrites exhibit high plasticity. Previous studies have demonstrated that severe stress reduces dendritic length and suppresses branching. To investigate the morphological basis of post‐intracerebral hemorrhage (ICH) depressive‐like behaviors and spatial learning/memory impairments, we evaluated dendritic complexity in prefrontal cortical neurons, pyramidal regions, and dendritic spine density in CA1 using Golgi staining. Dendritic spines, small protrusions extending from dendrites, serve as primary locations for establishing synaptic connections among neurons. In the CUMS rat model, a notable reduction in mushroom‐shaped spines and a decrease in spine thickness were observed compared to control animals. Notably, OPN treatment effectively counteracted these ICH‐induced alterations (Figure 8A–F). These results demonstrate OPN's capacity to mitigate ICH‐related changes. Furthermore, consistent with the observed improvements in dendritic spine density, the expression of PSD95 (Figure 6I,J), a critical postsynaptic marker, was significantly reduced in the ICH group and was notably rescued by OPN treatment (*p* < 0.01, ICH vs. Sham; *p* < 0.05, ICH + OPN vs. ICH; *n* = 3 per group).

## 4 Discussion

The findings of this study significantly advance our understanding of the pathophysiological mechanisms underlying post‐ICH depression and highlight the therapeutic potential of OPN modulation. The observed improvements in neurological function and depressive‐like behaviors following OPN administration in ICH model mice provide compelling evidence for its neuroprotective and mood‐regulating properties. These effects appear to be mediated through multiple interconnected pathways, including the modulation of neuroinflammatory responses, enhancement of neurotrophic support, and promotion of synaptic plasticity.

The anti‐inflammatory properties of OPN are particularly noteworthy in the context of post‐ICH depression. Following hemorrhagic stroke, the brain undergoes a complex cascade of inflammatory events characterized by microglial activation, cytokine release, and blood–brain barrier disruption [31]. Our data suggest that OPN may attenuate this neuroinflammatory milieu, thereby reducing secondary neuronal damage and creating a more favorable environment for neural repair. This anti‐inflammatory action is complemented by OPN's ability to upregulate BDNF expression, a key neurotrophic factor implicated in synaptic resilience, neurogenesis, and mood regulation. The synergistic interaction between these pathways likely contributes to the observed improvements in both neurological and behavioral outcomes.

However, the therapeutic application of OPN in post‐ICH depression is complicated by its context‐dependent and pleiotropic nature. While our findings demonstrate beneficial effects in the acute to subacute phases following ICH, it is crucial to recognize that OPN's biological activities may vary depending on the temporal stage of injury, cellular microenvironment, and receptor expression patterns. For instance, in certain pathological conditions, OPN has been reported to exert pro‐inflammatory effects or contribute to glial scar formation, potentially impeding neural repair [13]. This dualistic nature underscores the importance of developing targeted delivery strategies to maximize therapeutic efficacy while minimizing potential adverse effects.

Several innovative approaches could be explored to address these challenges. First, the development of nanoparticle‐based delivery systems could enable precise spatiotemporal control of OPN administration, allowing for targeted modulation of specific brain regions or cell populations. Second, the use of conditional knockout models could help elucidate the cell type‐specific functions of OPN in the context of post‐ICH depression. Third, combination therapies that integrate OPN modulation with established antidepressant treatments or neurorehabilitation strategies might offer synergistic benefits while reducing the required dosage of each individual component.

From a translational perspective, several critical steps are necessary to advance OPN‐based therapies toward clinical application. Comprehensive pharmacokinetic studies are needed to determine optimal dosing regimens and administration routes. Long‐term safety assessments should evaluate potential off‐target effects and systemic consequences of OPN modulation. Additionally, the development of reliable biomarkers for monitoring therapeutic response and disease progression would facilitate personalized treatment strategies.

The potential clinical implications of OPN modulation extend beyond post‐ICH depression. Given its involvement in various neuropsychiatric disorders and neurodegenerative conditions, insights gained from this research could inform therapeutic development for a broader range of neurological diseases. Furthermore, understanding the molecular mechanisms underlying OPN's effects on neuroinflammation and neuroplasticity may reveal novel therapeutic targets for mood disorders and cognitive impairment associated with brain injury.

Furthermore, while our bioinformatics analysis identified a network of 14 hub genes centered on SPP1, including key interactors like CD44 and ITGB2, this study focused on validating the therapeutic potential of the central node, SPP1/OPN. The contributions of these associated receptors and signaling molecules to the observed phenotype remain to be determined and represent a critical avenue for future research to fully delineate the downstream mechanistic pathway.

This study primarily focused on validating the therapeutic effects of OPN, and did not perform a correlation analysis between hematoma characteristics and behavioral manifestations. It is noteworthy that through standardized modeling procedures and random grouping, we ensured comparability of initial injury between groups, providing a reliable basis for therapeutic effect evaluation. Subsequent studies will incorporate imaging techniques to quantitatively analyze lesion characteristics and further investigate their correlation with behavioral performance.

A limitation of our experimental design is the absence of a control group that received OPN treatment without ICH induction. While our sham group provides a baseline for normal behavior, the OPN‐alone group would have allowed us to definitively rule out any intrinsic effects of OPN on motivation, locomotor activity, or baseline mood in healthy animals. Future studies incorporating such a control group will be valuable to confirm the context‐dependent therapeutic profile of OPN.

While our findings highlight the neuroprotective and mood‐stabilizing benefits of OPN in the context of post‐ICH depression, it is crucial to acknowledge its well‐documented dualistic, context‐dependent roles in other pathologies. Notably, OPN is frequently overexpressed in various cancers and is associated with promoting tumorigenesis, metastasis, and chemoresistance [13]. This potential pro‐tumorigenic risk, though likely minimal in the acute treatment setting for stroke, underscores the necessity for cautious long‐term safety evaluation when considering OPN‐based therapies.

To mitigate potential off‐target effects and maximize therapeutic efficacy, future clinical translation of OPN would greatly benefit from the development of advanced, targeted delivery systems. Strategies such as cell‐penetrating OPN peptide fragments, OPN‐loaded nanoparticles functionalized with brain‐targeting ligands, or even gene therapy approaches using cell‐type‐specific promoters could enable precise spatiotemporal delivery of OPN to the injured brain regions. This would maximize local therapeutic concentration while minimizing systemic exposure and potential unintended consequences on peripheral tissues.

In conclusion, this study establishes OPN as a promising therapeutic target for post‐ICH depression, with demonstrated neuroprotective and mood‐stabilizing properties. The multifaceted nature of OPN's biological activities presents both opportunities and challenges for therapeutic development. Future research should focus on elucidating the precise molecular mechanisms of OPN's actions, optimizing delivery strategies, and evaluating clinical feasibility through well‐designed translational studies. The integration of OPN modulation into comprehensive treatment paradigms could ultimately improve outcomes for patients suffering from this debilitating complication of hemorrhagic stroke, while also advancing our understanding of the complex interplay between neuroinflammation, neuroplasticity, and mood regulation in brain injury.

## Author Contributions

Pengpeng Li and Yangyang Gao designed and performed the experiments, and wrote the manuscript. Shiqing Du and Zhengqian Mu analyzed the data. Zhenxing Tao and Xuqi Zhang conducted the statistical analysis. Xudong Zhao conceived the study, supervised the project, and revised the manuscript. All authors reviewed and approved the final manuscript.

## Funding

This study was supported by the National Natural Science Foundation of China (grant no. 82071381 awarded to Xudong Zhao).

## Ethics Statement

All animal experiments were conducted in accordance with the National Institutes of Health Guide for the Care and Use of Laboratory Animals and were approved by the Animal Ethics Committee of The Affiliated Wuxi No. 2 People's Hospital (Approval No. 2023Y202). The study is reported in accordance with ARRIVE guidelines.

## Conflicts of Interest

The authors declare no conflicts of interest.

## Supporting information


**Figure S1:** Comparison of mortality rates between control and OPN‐treated ICH groups.

## Data Availability

The datasets generated and/or analyzed during the current study are available from the corresponding author on reasonable request.
