# Supplementary material for: SPP1/OPN Alleviates Post‐Intracerebral Hemorrhage Depression and Cognitive Impairment via Nrf2/BDNF Signaling Activation in Mice
Source: CNS Neurosci Ther. 2025 Dec 4;31(12):e70680. doi: 10.1002/cns.70680 (PMC12678055; doi:10.1002/cns.70680)
Supplement: Supplementary file 1 — Figure S1: Comparison of mortality rates between control and OPN‐treated ICH groups. [file CNS-31-e70680-s001.docx]

**Supplementary Figure 1: Comparison of Mortality Rates Between Control and OPN-Treated ICH Groups**


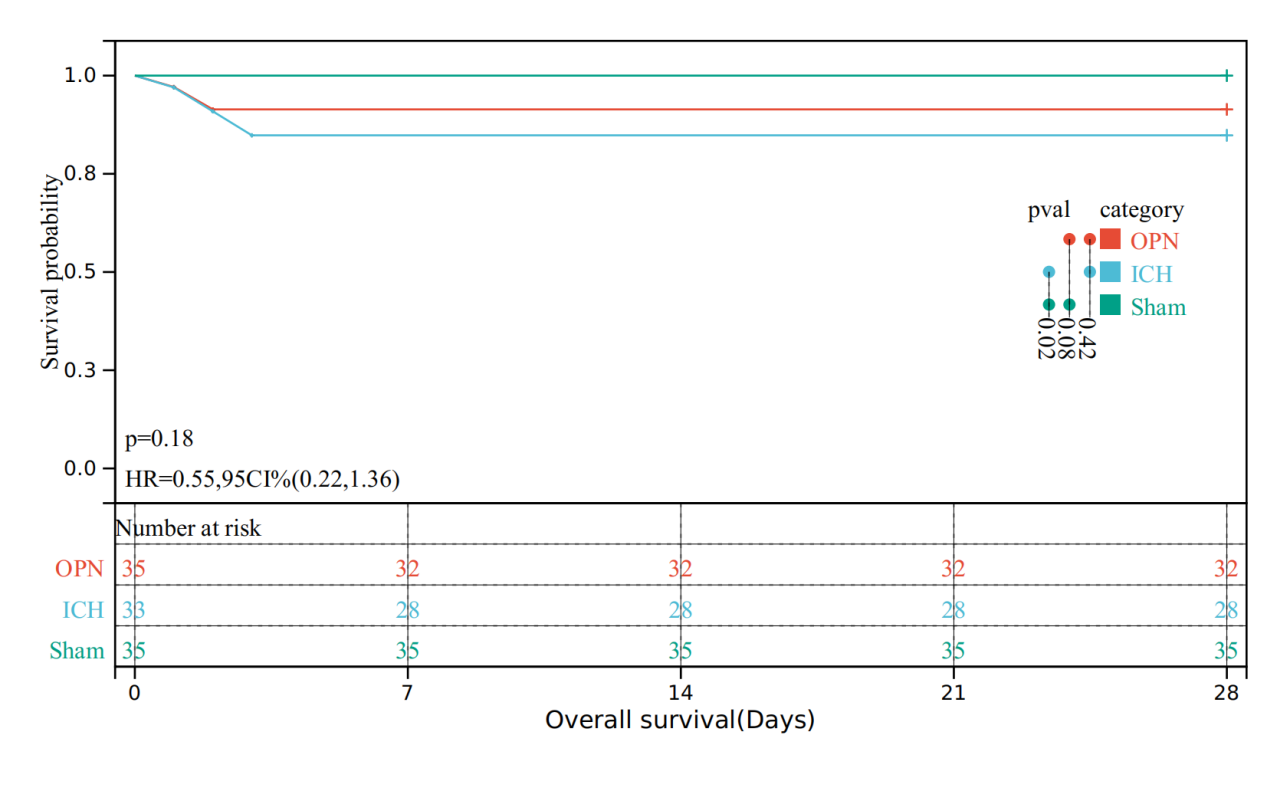


The figure illustrates the mortality rates in the control intracerebral hemorrhage (ICH) group (15.1%, 5/33) and the OPN-treated ICH group (8.5%, 3/35).
